# A scalable route to quaternary ammonium-functionalized AgCl colloidal antimicrobials inhibiting food pathogenic bacteria and biofilms

**DOI:** 10.1016/j.heliyon.2024.e25260

**Published:** 2024-02-01

**Authors:** Diellza Bajrami, Syed Imdadul Hossain, Alexia Barbarossa, Maria Chiara Sportelli, Rosaria Anna Picca, Luigi Gentile, Francesco Mastrolonardo, Antonio Rosato, Alessia Carocci, Nicola Antonio Colabufo, Boris Mizaikoff, Nicola Cioffi

**Affiliations:** a Institute of Analytical and Bioanalytical Chemistry, Ulm University, Albert Einstein-Allee 11, 89081, Ulm, Germany; b Chemistry Department, University of Bari “Aldo Moro”, Via E. Orabona, 4, 70126, Bari, Italy; c CSGI (Center for Colloid and Surface Science) C/o Dept. Chemistry, Via E. Orabona, 4, 70126, Bari, Italy; d Department of Pharmacy-Drug Sciences, University of Bari “Aldo Moro”, 70126, Bari, Italy; e Biofordrug Srl, University of Bari “Aldo Moro”, Via Dante 95, 70019, Triggiano, Bari, Italy; f Hahn-Schickard, Sedanstrasse 14, 89077, Ulm, Germany

**Keywords:** Nanoantimicrobials, Colloids, AgCl, QACs, Antibacterial, Antibiofilm

## Abstract

This study explores how a simple argentometric titration-like approach could be evolved into a versatile, scalable, fast, and robust strategy for the production of AgCl/quaternary ammonium compounds (QACs) colloidal nanoantimicrobials (NAMs). These systems, which are green, stable, cost-effective, and reproducible are found to be effective against a wide range of food pathogenic bacteria and biofilms. The option of a large-scale production for such colloidal suspensions was explored via the use of a peristaltic pump. The utilization of various types of biosafe QACs and a wide range of solvents including aqueous and organic ones renders this system green and versatile. Nanocolloids (NCs) were characterized using UV–Vis, X-ray photoelectron and Fourier transform infrared (FTIR) spectroscopies. Their morphology and crystalline nature were investigated by transmission electron microscopy (TEM) and selected area diffraction pattern (SAED). Nanoparticle (NP) size distribution and hydrodynamic radius were measured by dynamic light scattering (DLS), while the ζ-potential was found to be highly positive, thus indicating significant colloidal stability and antimicrobial activity. In fact, the higher the NP surface charge, the stronger was their bioactivity. Furthermore, the antibacterial and antibiofilm effects of the as-prepared NCs were tested against Gram-positive bacteria, such as *Staphylococcus aureus* (ATCC 29213) and *Listeria monocytogenes* 46*,* and Gram-negative bacteria, such as *Escherichia coli* (ATCC 25922) and *Pseudomonas aeruginosa* (ATCC 27853)*.* The results clearly indicate that AgCl/QACs provide pronounced antibiofilm activity with long-term bacteriostatic effects against foodborne pathogenic bacteria rendering them an ideal choice for active food packaging systems.

## 1 Introduction

Biofilm science has been an active field of study at the single-cell level, as the majority of microorganisms adhere to solid surfaces. Microbial biofilms are bacterial associations composed of multicellular cells embedded in the self-produced extracellular polymeric substances (EPS) matrix of the bacterial community [1]. Biofilms present challenges and difficulties in food industry and healthcare systems, owing to their fast attachment on food manufacturing surfaces [2,3]. Once a biofilm's tridimensional structure is strongly formed, the complexity of the ecosystem becomes noticeable for manual removal. The biggest concern is coming from the human pathogenic bacteria which are biofilm-forming species colonizing the food processing contact like mixing tanks, drains, pipes and other surfaces, made of stainless steel, polyethylene, wood, glass, rubber, plastic, etc. [4,5]. Foodborne pathogens that cause cross-contamination of food products represent a serious problem for food safety and quality in today's globalizing market for raw food [6,7]. Many bacterial species of food contaminants can exist in the same habitat and interact to form multispecies biofilms [8]. Microbial interactions between different persistent bacterial strains in food-related environments are difficult to control [9]. Foodborne illnesses associated with the consumption of contaminated dairy products, vegetables, and fruit, have increased significantly with the emerging requirement of research in this direction [10,11]. *Listeria monocytogenes* is one of the most dangerous Gram-positive pathogenic bacteria for human health, causing listeriosis by consuming contaminated food [12]. A foodborne disease outbreak still present nowadays, with a high mortality rate, indicates an urgent warning emphasizing the risk of contaminated products [13]. *Staphylococcus aureus* is one of the most common food-poisoning microorganisms isolated from fresh and processed food [14,15]*.* The ability of these pathogenic bacteria to adhere to the biofilm structure enhances their continuous microbial infection during food processing, storage, and distribution [16]. The acquisition of antibiotic resistance and the tendency to form robust biofilms is an ability of the Gram-negative bacterium *Pseudomonas aeruginosa* [17]. Most antibiotics are unable to eradicate adherent microbial cells in biofilm structure of this pathogenic microorganism [18]. Food alteration and contamination by spoilage *Pseudomonas aeruginosa* are found in fruits, vegetables, pasteurized milk products, and meat surfaces [19]. Serious large-scale food poisoning cases also arise from the Gram-negative bacterium *Escherichia coli,* which is one of the strongest antibiotic-resistant bacteria, causing diseases ranging from mild diarrhea to hemorrhagic colitis [20]. Sessile communities of *E. coli* biofilms can be formed at various temperatures relevant for food production, handling and distributing, leading to cross-contamination of food products [21,22].

Antibiotic-resistant bacteria reach the food chain by consuming contaminated food in contact with colonized surfaces [23]. Despite the improved cleaning and disinfection monitoring strategies, biofilms persistently resistant to antimicrobials (e.g., antibiotics, biocides, etc.) remain the same ubiquitous problem [24,25]. Microbial biofilms have up to 10000-fold more antibiotic resistance than planktonic forms, because changes in the biofilm structure trigger the accumulation of metabolites, which are responsible for the dynamics between bacterial species and impact antibiotic susceptibilities [26,27].

Nanocolloids (NCs) and nanoparticles (NPs) are increasingly being applied to target pathogenic biofilm-producing bacteria as alternatives to traditional antibiotics [[28], [29], [30]]. It is known that Ag-based nanocomposites have effective antimicrobial and antibiofilm properties against a wide range of viruses, fungi, algae, and bacteria, and low cytotoxicity towards mammals [31]. Among the metal-based nanoantimicrobials, AgNPs are the most commonly used materials in consumer products, cosmetics, biomedical, and pharmaceutical fields, not only for the delivery of therapeutic agents, but also for inhibiting the growth of pathogenic bacteria [32]. Depending on the size, shape, concentration, solubility, surface charge, silver ion release properties, complexation with various ligands (chloride, sulfide, thiosulfate), complexation with conventional antibiotics, AgNPs have shown numerous mechanisms of action against bacteria. The most likely mechanisms are: (i) the interaction of NPs with bacterial cell membranes; (ii) the release of silver ions; (iii) the simultaneous generation of ROS and free radicals, which lead to bacterial cell injury or death [33]. To obtain long-term antimicrobial effects and reduce toxicity, a controlled and slow release of silver ions is required. From a cytotoxicity perspective, silver halides are safer than AgNPs. They have a very low solubility, which ensures the release of a low and constant concentration of bioactive Ag^+^ ions and increases the shelf-life of food products. A milestone in this area is represented by the seminal paper by V. Sambhy et al. on AgBr-based nanoantimicrobials [34]. In the following years, AgCl has also received attention as antimicrobial agent [[35], [36], [37]]. Different approaches, including photochemistry, electrospinning, thermal decomposition, and radiation have been used for the production of AgCl [38,39]. Nonetheless, some of these methods require multiple steps, sophisticated equipment, or the use of toxic chemicals, and are mostly limited to lab-scale production. However, in view of the rapid, large-scale production of antimicrobial agents, green character, cost-effectiveness, scalability, and versatility should be considered. We recently investigated the addition of antimicrobial QAC, e.g., benzalkonium chloride (BAC) with AgCl, to improve the stability of NCs, provided that the aromatic BAC moieties act as a bilayer around AgCl NPs, forming a core-shell structure [40]. However, in that preliminary study we did not investigate the potential of these nanocolloids as synergistic antimicrobial or antibiofilm agents.

This research field is therefore mature for a further step, aimed at deepening the knowledge on the versatility, scalability, and robustness of silver halide-based systems. With the present study, we aim to extend the titration-based route to AgCl-based antimicrobials involving different QACs, prove the method scalability and versatility, and demonstrate its synergistic potential in antibiofilm applications.

Different QAC chlorides are already diffused at the industrial level and can be suitable candidates as silver halide capping agents. The biodegradable and disinfecting agent dodecyl-dimethyl-ammonium chloride (DDAC) has been approved by the European Union as a biocidal agent [41]. On the dietary risk assessment, temporary maximum residual level proposed by European Food Safety Authority (EFSA) for DDAC is 0.1 mg/kg. These values are considered to be sufficiently protective for food consumers [42]. DDAC has already proven to be effective against bacteria by disrupting the cell membrane, resulting in the leakage of intracellular molecules and cell injury [43]. Nonetheless, the use of DDAC alone can decrease antimicrobial susceptibility [44]. The role of dimethyl-octadecyl-ammonium chloride (DDoAC) in racing antimicrobial resistance also needs to be investigated. DDAC and DDoAC surfactants should have an intimate relationship to control the morphology of metal NPs [45]. The selection of bioactive and safe DDAC and DDoAC for the production of synergistic AgCl could open new perspectives in combacting pathogenic microbials and biofilms. In the present study, a simple titration-like approach using a peristaltic pump was applied for the large-scale production of AgCl/DDAC and AgCl/DDoAC NCs. As for the AgCl/DDAC NCs, an aqueous medium was employed, while AgCl/DDoAC NCs were produced in isopropanol (IPA). In all cases, 100% titration was chosen, to ensure the maximum yield possible. To identify the best antimicrobial and antibiofilm agent, both AgCl/DDAC and AgCl/DDoAC NCs were critically compared from a materials science and biological efficiency point of view. The antibacterial and antibiofilm activities of both the samples were evaluated against *Staphylococcus aureus* ATCC 29213, *Listeria monocytogenes* 46, *Escherichia coli* ATCC 25922, and *Pseudomonas aeruginosa* (ATCC 27853). AgCl/DDAC was found to strongly inhibit *Staphylococcus aureus* ATCC 29213 *an*d *Escherichia coli* ATCC 25922. AgCl/DDoAC is considered the best candidate for *Listeria monocytogenes* and Pse*udomonas aeruginosa* (ATCC 27853). Notably, both candidates were found to be potential inhibitors of the tested food-pathogenic bacteria and biofilms.

## 2 Materials and methods

### 2.1 Materials

Silver nitrate (AgNO_3_, 169.87 g/mol; 99.99% purity) and DDoAC (586.50 g/mol; ≥97%) were purchased from Sigma–Aldrich (Milan, Italy). DDAC (362.08 g/mol; ≥95%) was purchased from Santa Cruz Biotechnology (Santa Cruz, CA, USA). Milli-Q water and isopropyl alcohol (IPA, anhydrous, 99.5%, Sigma Aldrich, Milan, Italy) were used throughout the experiments.

### 2.2 Synthesis of AgCl/DDAC and AgCl/DDoAC NCs using peristaltic pump

**Fig. 1:**
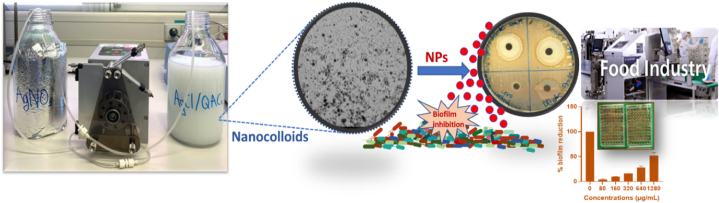
Schematic representation of the production of AgCl/QAC nanoantimicrobials using a peristaltic pump and their potential application in food industry.

Stock solution of 50 mM AgNO_3_/Milli-Q, AgNO_3_/IPA, DDAC/Milli-Q, and DDoAC/IPA were prepared for the experiments. The experimental setup is illustrated in Fig. 1. NCs of AgCl/DDAC were prepared by mixing AgNO_3_ and DDAC aqueous solutions.

In brief, 500 mL of AgNO_3_ (50 mM) titrating solution were added to 500 mL of DDAC (50 mM) solution to produce AgCl/DDAC (1:1) NCs. Following the similar approach, AgCl/DDoAC (1:1) was prepared by mixing 500 mL AgNO_3_ (50 mM) and 500 mL DDoAC (50 mM) solutions using IPA as solvent. In both experiment the mixing occurred using a peristaltic pump (0.78 mL/min). A milky-light color was visible upon the formation of AgCl. After the dropwise addition of 500 mL of AgNO_3_ to 500 mL of QACs solution, the reaction mixture was kept under stirring for a minimum of 10 min before carrying out further characterization and biological applications. AgCl/DDAC and AgCl/DDoAC as powder form can be achieved by centrifugation, and subsequent drying at room temperature.

### 2.3 UV–Vis and XPS characterization

UV–Vis spectroscopy characterization of AgCl/DDAC and AgCl/DDoAC NCs was performed using Shimadzu UV-1601 double beam spectrometer; all spectra were operated between 200 and 500 nm range, in with 1-cm quartz Suprasil® cuvettes from Hellma Analytics (Müllheim, Germany). X-ray photoelectron spectroscopy (XPS) measurements were performed using a PHI 5000 Versa Probe II Scanning XPS Microprobe spectrometer (ULVAC-PHI Inc., Kanagawa, Japan). The measurements were performed with a monochromatized Al Kα source (1486.6 eV, X-ray spot 200 μm), at a power of 50.3 W. Dual-beam charge neutralization was constantly applied during the analysis. Wide scans and detailed spectra were acquired in Fixed Analyzer Transmission (FAT) mode with a pass energy of 117.40 eV and 46.95 eV, respectively. An electron gun was used for charge compensation (1.0 V, 20.0 μA). The Binding Energy (BE) scale was calibrated by fixing the aliphatic component of the C1s signal (BE = 284.8 ± 0.2 eV) as a reference. Spectral processing was performed using MultiPak software v. 9.9.0.8. The modified Auger parameter (α′) was calculated as the sum of the BE (Ag3d_5/2_) and the kinetic energy (KE) of the AgM_4_N_45_N_45_ sharpest peak [46].

### 2.4 FTIR characterization of silver chloride nanocolloids

The IR spectra of the dried AgCl/DDAC and AgCl/DDoAC samples were obtained using a single-bounce diamond attenuated total reflectance (ATR) assembly (Platinum ATR, Bruker Optics, Ettlingen/Germany) and a Bruker Alpha II FTIR spectrometer. Prior to recording the spectra, the nanocolloidal samples were deposited onto the ATR crystal, collecting air as the background spectrum. Each IR spectrum represents an average of 64 scans at a spectral resolution of 2 cm^−1^, in the spectral range 4000–400 cm^−1^, using OPUS software (Bruker Optics, Ettlingen/Germany). Spectra were batch converted and processed using the Essential FTIR software (Operant LLC, Madison/USA).

### 2.5 TEM and DLS characterization

TEM was performed using a FEI Tecnai 12 instrument (120 kV; filament: LaB_6_). AgCl/DDAC NCs were drop-cast onto copper grids (300 mesh, Agar Scientific) and AgCl/DDoAC NCs onto carbon grids (300 mesh, Agar Scientific) in volumes of 3–5 μL for each sample. Dynamic light scattering (DLS) measurements were performed using a Zetasizer Nano ZS instrument (Malvern Instruments, Ltd., Worcestershire, UK) as described previously [40]. The Zetasizer Nano ZS is equipped with a 4 mW He−Ne laser and an automatic laser attenuator, and with an avalanche photodiode detector. All measurements were recorded at the scattering angle, θ, of 173°, as well as zeta-potential measurements (at θ = 12.8°). The temperature was set to 25 °C. The hydrodynamic radius (RH) was determined using the Stokes−Einstein equation. Both 20 mM and 50 mM of AgCl/DDAC and AgCl/DDoAC samples were subjected to TEM and DLS characterization. Furthermore, solo 20 mM AgCl/DDoAC samples were filtered using 45 μm filter followed by DLS and TEM characterization.

### 2.6 Bacterial strains, isolation and culture conditions

The foodborne pathogenic strains used in this study were Gram-positive bacteria *Staphylococcus aureus* ATCC 29213 and *Listeria monocytogenes* 46, and Gram-negative bacteria *Pseudomonas aeruginosa* ATCC 27853 *and Escherichia coli* ATCC 25922. *S. aureus*, *P. aeruginosa* and *E. coli* were obtained from the American Type Culture Collection (ATCC, Rockville, MD, USA), whereas *L. monocytogenes* 46 was isolated from clinical samples. The strains were stored as stock solutions in glycerol at −80 °C. Mueller-Hinton agar (MHA) was used for disk diffusion test culture media plates, and Mueller-Hinton broth (MHB) and Tryptic Soy Broth (TSB+ 2% glucose) was used for antimicrobial and antibiofilm testing. The incubation temperature for all the bacterial cultures was 37 °C, for 24 h.

### 2.7 Agar well disk diffusion test and comparison to conventional antibiotics

The agar disk-diffusion technique was used to determine the antibacterial performance of AgCl/DDAC and AgCl/DDoAC compared to conventional antibiotics, and their efficiency against four food pathogenic bacteria listed above. The assays were performed according to the methodology recommended by the Clinical and Laboratory Standard Institute (CLSI) and M-44A protocol [47]. This method has been used to evaluate the antimicrobial activity of NC solutions by determining their zone of inhibition.

Briefly, Petri dishes with MHA (Muller Hinton Agar, Oxoid) agar were inoculated with liquid bacterial suspensions (using Q-Tips in plates). Sterilized paper discs were placed on agar plates, and 25 μL of antimicrobial solution was poured on top of the discs. Four discs were used for the analysis: one for freshly prepared NCs (AgCl/DDAC or AgCl/DDoAC), one for a 3-week-old NC sample (to assess stability towards aging), and one for the control (i.e., surfactant used for the synthesis: DDAC and DDoAC). The 4th disc was a control of 5 μg Oxoid™ Levofloxacin Antimicrobial Susceptibility, as a conventional antibiotic. The plates were then incubated at 37 °C for 24 h. Subsequently, the zone of inhibition of antimicrobial activity was evaluated.

### 2.8 Microdilution antimicrobial susceptibility test by determination of MICs

The minimum inhibition concentration (MIC) against the four food pathogenic strains was followed by using the guidelines of the CLSI procedure of broth microdilution methodology outlined in ISO 20776-1 (CLSI M7A9 protocol) [48]. MIC values were determined by the microdilution method in 96-well microtiter plates using nanocomposite systems in concentrations derived traditionally from serial two-fold dilutions indexed in base 2. Overnight bacterial cultures were diluted at the controlled values OD_625nm_ = 0.08–0.1 and were used in the presence and absence of silver chloride NCs. For the intermediate (10x) antimicrobial solutions, the dilution of the concentrated antimicrobial stock solutions was performed. Briefly, 100 μL of sterile MH broth were added to each well of a microtiter plate. The most convenient method for preparing microdilution trays is the use of a dispersing device and antimicrobial dilutions in at least 10 mL of broth. 100 μL of AgCl/DDAC or AgCl/DDoAC NCs were mixed with 9.9 mL of MHB medium. Each tray included a growth control well and a sterile (uninoculated) well. Three controls were included in the study: (i) positive control with the inoculum only; (ii) negative control containing Muller–Hinton broth, Oxoid medium; (iii) Gentamicin antibiotic 64 μg/mL performed in duplicates (each by addition of 100 μL Gentamicin solution into microtiter wells). Using a multichannel micropipette 200 μL of AgCl/DDAC was added to duplicate wells and serially diluted to four additional wells. The same procedure was followed for the AgCl/DDoAC noncolloidal system. Two duplicates were performed for each strain. Then, 100 μL of inoculated bacteria in 9.9 mL media was serially added to the wells specific to four different strains. The same protocol was followed for an additional microtiter plate containing surfactants DDAC and DDoAC. The active compounds in the culture plates were incubated at 37 °C for 24 h, to find out antibacterial activity. The growth of microorganisms was evaluated based on the turbidity and pellets formed at the bottom of the well.

### 2.9 Antibiofilm activity of AgCl noncolloidal suspensions

To reveal the cell vitality of the strains treated with the products, a mixture of XTT-menadione was used. Living bacterial cells can reduce tetrazolium salt (2.3-Bis(2-methoxy-4-nitro-5-sulfophenyl)-2H- tetrazolium-5carboxanilide, XTT) in a hydro soluble composite called formazan, owing to NADH-mitochondrial dehydrogenases of the electron transport system (ETS) using an artificial electron acceptor (redox pigment) that competes with oxygen [49]. The evaluation of the optical density revealed by the action of AgCl/DDAC and AgCl/DDoAC NCs against biofilm growth was measured for the four pathogenic strains tested. Details of the formazan formation reaction from XTT mixture, culture phase and maturation phase are shown as Supplementary Information (SI).

### 2.10 Colony forming unit (CFU) for cell viability

Viability reduction of the bacterial strains tested against nanocolloidal systems was performed using the viability assay method. Glass coverslips and silicon surfaces (cut into square shapes of approximately 1 cm^2^ from the original 10 cm diameter wafers) were rinsed with acetone, 2-propanol, and deionized water to remove organic contamination, and subsequently sterilized by autoclaving. The coverslips were covered with 500 μL of AgCl/DDAC and AgCl/DDoAC nanocolloidal solutions and dried overnight. The modified coverslips were tested for antimicrobial activity after incubation at 37 °C for 24 h, and the efficiency against the four pathogenic strains was confirmed by OD measurements. The diluted aggregated cells left in 15 mL Eppendorf tubes were homogenized by short vertexing, and the resulting cultured suspension (100 μL) was added to 9.9 mL of sterile PBS (pH = 7.1). Then, a 1 mL aliquot was serially diluted 10 folds (CLSI M7A9). 100 μL aliquot from each dilution was aseptically withdrawn, spread on MHI agar plates, and incubated at 37 °C for 24 h before viable counts were determined.

## 3 Results and discussion

### 3.1 Characterization of silver chloride NCs

**Fig. 2:**
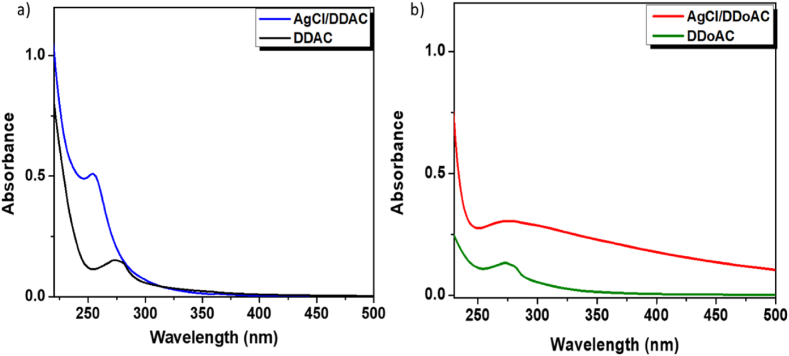
UV–Vis absorption spectra of (a) AgCl/DDAC and DDAC, and (b) AgCl/DDoAC and DDoAC.

AgCl (nano)-particles have peculiar absorption in the UV–Vis range between 250 and 290 nm, depending on the eventual stabilizing molecule. Hence, UV–Vis absorption spectra of AgCl/DDAC and AgCl/DDoAC provide preliminary information of the formation of AgCl, as shown in Fig. 2. The colloidal dispersion of AgCl/DDAC (Fig. 2a) exhibited a strong and intense peak at 255 ± 2 nm, which could be attributed to surfactant-coated AgCl [[50], [51], [52], [53]] The same could be argued for AgCl/DDoAC (Fig. 2b).

An absorption peak at 275 ± 2 nm was observed for the solo DDAC aqueous solution. This peak is not observed in AgCl/DDAC, possibly because of the lower amount of free DDAC molecules in solution, most of the quaternary ammonium species being expected to be located around AgCl NPs. Involving surfactant molecules, corresponding peak absence in the fingerprint region of NPs spectrum can be observed. This absence strongly supports the fact that specific groups are intricately involved in both the synthesis and stability of nanoparticles [54,55]. The DDoAC/Isopropanaol (IPA) solution alone exhibited a peak at 273 ± 2 nm. Meanwhile, the colloidal dispersion of AgCl/DDoAC showed a very broad band around 260–350 nm, possibly because of the overlap of spectral features due to stable AgCl formations, in the presence of DDoAC multilamellar structures (MLS) [40,56].

It is known that AgCl NPs have high direct and indirect band gap, showing signal in the ultraviolet region (200–300 nm) [57]. Direct band gap for AgCl/DDAC and AgCl/DDoAC was calculated to be 5.5 eV and 5.3 eV respectively, as shown in Fig. S1. These values are in agreement with the values of the AgCl NPs band gap reported in the literature [58,59] and the systems possibly prevent AgCl from absorbing visible light [53]. AgCl does not exhibit a surface plasmon resonance peak (SPR) around 400 nm, which implies that the used surfactant prevents the photo-reduction of AgCl to Ag (0) thanks to a chemical stabilization operated by DDAC and DDoAC.

**Fig. 3:**
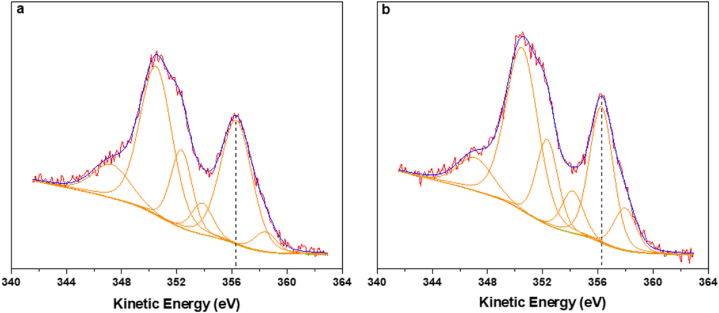
AgM_4,5_N_45_N_45_ Auger spectra of AgCl/DDAC (a) and (b) AgCl/DDoAC.

XPS characterization was performed on AgCl/DDAC and AgCl/DDoAC to confirm the formation of AgCl in the colloidal suspension. The binding energy (BE) position of Ag3d_5/2_ centered at 367.1 ± 0.3 eV and 367.5 ± 0.2 eV (Fig. S2) for AgCl/DDAC and AgCl/DDoAC respectively, are compatible with the existence of AgCl in both systems [60,61]. However, the limited chemical shift typically observed on silver photoelectron signal induced us to confirm attribution to AgCl by recording and processing AgMNN Auger signals (Fig. 3a and b). In fact, the modified Auger parameter (α′), given by the sum of BE(Ag3d_5/2_) and kinetic energy maximum (KE_max_) of AgM_4,5_N_45_N_45_, is a better diagnostic tool for silver speciation. The estimated α′ value was 723.4 ± 0.3 eV and 723.7 ± 0.3 eV for AgCl/DDAC and AgCl/DDoAC, respectively, which is in good agreement with 723.5 eV, reported for AgCl in Ref. [62]. Values typically observed for Ag_2_O are in the range 724.2–724.4 eV [62,63]. Moreover, the Cl/Ag elemental ratio found in the XPS quantitative analysis of both samples agrees well with the stoichiometric value, being 0.97 ± 0.07.

**Fig. 4:**
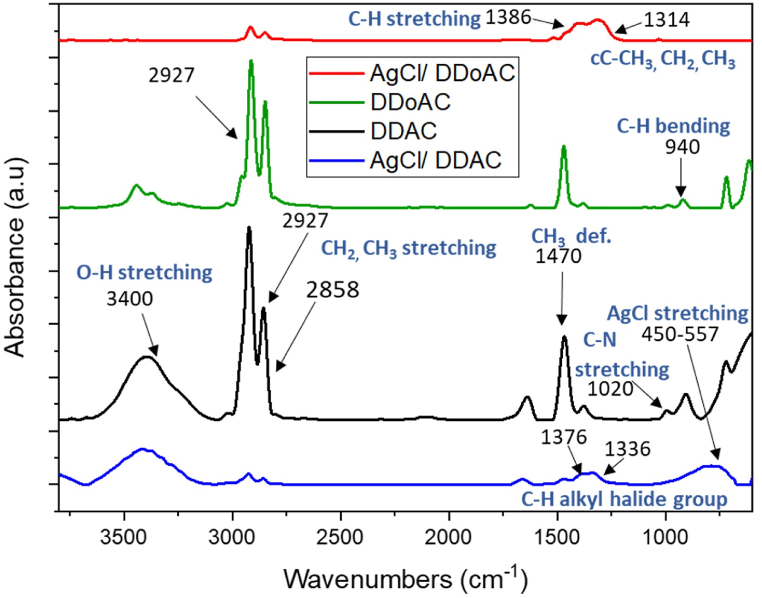
ATR-FTIR spectra of AgCl/DDAC and AgCl/DDoAC as compared to DDAC and DDoAC spectra. Main assignments are highlighted.

The synthesis of silver chloride nanocolloids in the reaction with DDAC and DDoAC was confirmed by ATR-FTIR spectroscopy. The ATR-FTIR spectra of AgCl/DDAC and AgCl/DDoAC compared to that of the surfactant itself are shown in Fig. 4.

FTIR analysis of AgCl NCs was carried out to identify the functional groups present in the AgCl/DDAC and AgCl/DDoAC NPs, as shown in Fig. 4. The broad band at 3400 cm^−1^ indicates the presence of a hydrogen bonded O–H peak, confirming that some water is trapped within the cationic surfactant molecule [64]. The bands at 2927 and 2858 cm^−1^ can be assigned to the –CH_3_, –CH_2_, and sp^3^ C–H stretching modes, respectively, indicating the presence of DDAC and DDoAC components acting as capping molecules in the AgCl NPs structure [64,65]. The peak at 1470 cm^−1^ represents CH_2_ bending deformation [53,65,66]. This particular moderate peak was attributed to the strong methylene vibrations in both samples, originating from the connection to the remaining amine groups in the DDAC and DDoAC molecular structures [67]. All peaks appear with similar absorbance intensities in the presence of AgCl nanoparticles formed, except for the peaks centered at 2927 and 2858 cm^−1^, which appear much lower in intensity, possibly due to the attachment of the surfactant at AgCl nanoparticles through van der Waals interactions. The peaks in the regions 1386 and 1376 cm^−1^ are characteristic of C–H alkyl halide groups, indicating that DDAC and DDoAC surfactant-positive ammonium ions and negative chloride anions are responsible for charge separation and react with silver ions to form AgCl. The peak at 1336 cm^−1^ is attributed to the silver halide chemical bond into the AgCl nanoparticle core. The small band at 1020 cm^−1^ is related to aliphatic C–N stretching vibrations [64], and that at 940 cm^−1^ (the presence of C–H out-of-plane, bending) arises from the cationic surfactants DDAC and DDoAC interacting with AgNO_3_ [67,68]. The infrared (IR) range and the strength of absorbance peak exhibit slight variations based on the specific structure of the other surfactant, DDoAC and its molecular environment [68]. The Ag–Cl stretching vibrations (alkyl halides) occurring at 450-557 cm^−1^ are influenced by factors such as particle size, shape, and the presence of surfactant agents [53,69].

### 3.2 DLS and TEM characterization

**Fig. 5:**
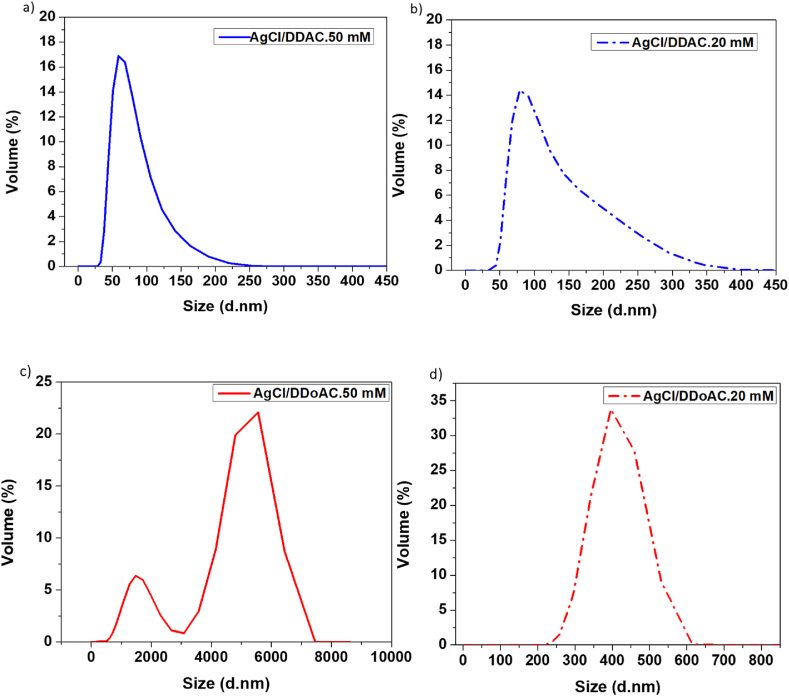
Size distribution by volume % of (a) AgCl/DDAC. 50 mM, (b) AgCl/DDAC. 20 mM, (c)AgCl/DDoAC. 50 mM and (d) AgCl/DDoAC. 20 mM (450 nm filtered).

**Fig. 6:**
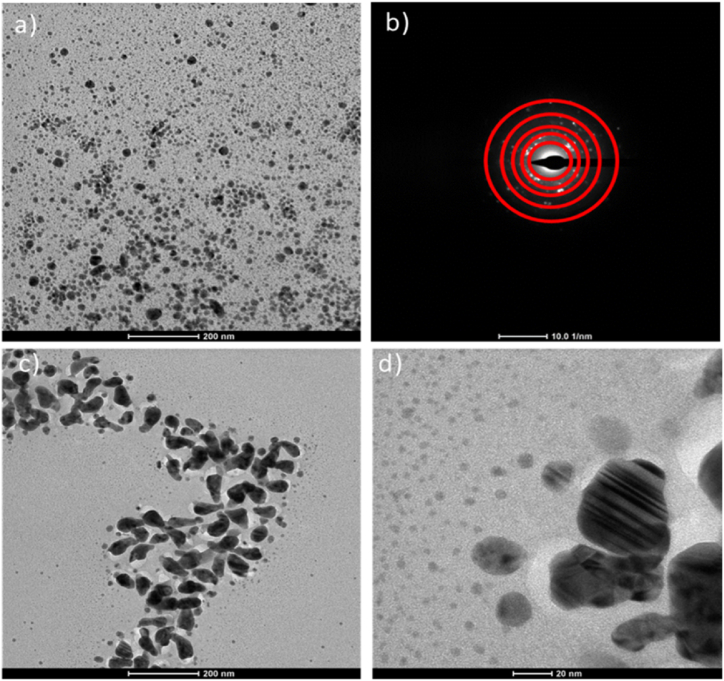
(a) Typical TEM images, (b) selected area electron diffraction pattern of AgCl/DDAC 50 mM, (c) and (d) Typical TEM images of AgCl/DDoAC 50 mM.

DLS measurements suggested the presence of surfactant molecules around the AgCl NPs. The larger diameter is due to the presence of surfactants micelles around the inorganic AgCl core as already explained in previous papers [40,56]. As an example, for the AgCl/DDAC sample, an average diameter of 65 ± 5 nm was observed by DLS (Fig. 5a), larger than the corresponding TEM size measurement, (Fig. 6a), and this is in agreement with the DLS mean diameter being 20–30% higher than the average TEM size [70]. In case of DDoAC, formation of stabilizer vesicles must be considered [56]: these vesicles surrounding AgCl clusters are responsible for the higher hydrodynamic radius registered.

The selection of QAC and its concentration are crucial for the preparation of stable colloidal particles. Therefore, DLS was performed at different concentrations. However, a considerable difference in size aggregates was observed between the AgCl/DDAC samples, Fig. 5a and b, and the AgCl/DDoAC ones, Fig. 5c and d. According to a previous study, DDoAX (X = Br or Cl) vesicles could appear as milky fluids in the range of 10–20 mM, and highly viscous milky dispersions above 40 mM concentration [71]. In addition, multilamellar structures (MLS) appear at concentrations between 1 mM and 65 mM [56]. DLS characterization was performed for both 50 mM and 20 mM AgCl/DDoAC. QAC can act as a stabilizer forming either micelles, as observed for the DDAC, or vesicles, as observed for the DDoAC, around the NPs. The schematic representation of AgCl nanocolloids stabilized by DDAC^+^ and DDoAC^+^ molecules is shown in Fig. S3.

The 50 mM AgCl/DDAC solution showed a very sharp peak in the volume-weighted size distribution at approximately 60 nm with a narrow size distribution, which indicates that the AgCl NPs were of uniform size. An average diameter of 65 ± 5 nm was observed by DLS (Fig. 5a). A zeta potential value of 58 ± 3 mV was found for AgCl/DDAC 50 mM system. As to the AgCl/DDAC 20 mM, sharp peak was obtained at 80 nm, with an average diameter of 85 ± 5 nm (Fig. 5b) and a zeta potential value of 50 ± 5 mV. These results demonstrate the formation of a DDAC layer around AgCl NPs, providing preliminary information on the possible long-term colloidal stability, and long-term antimicrobial activity, considering that high positive zeta potential values are sufficient to prevent colloids aggregation and cause NPs to be more lethal to bacteria [72,73].

Owing to the presence of DDoAC multilamellar vesicles, a wide size distribution curve is reasonable for the AgCl/DDoAC 50 mM colloids, as shown in Fig. 5c. Hence, 450 nm filtered AgCl/DDoAC 20 mM was used to provide information on the formation of AgCl (Fig. 5d). A narrower size distribution, a sharp peak at 395 nm, and an average diameter of 500 ± 90 nm were obtained.

TEM provided further insight on the NPs morphology. Typical TEM images of AgCl/DDAC are shown in Fig. 6a, which shows spheroidal nanoparticles with an average diameter of 15 ± 5 nm. In a previous study based on the asymmetric benzalkonium chloride capping agent [40], a decreased stability was noted at the complete stoichiometric titration, due to the inability of surfactant molecules to provide sufficient protection. Nonetheless, a complete stoichiometric reaction is required for gaining the maximum yields, in conformity to the green chemistry principle of atom economy. A strong and symmetric QAC surfactant, such as DDAC, could allow overcoming this limitation.

Fig. 6b shows a selected area electron diffraction pattern (SAED) of AgCl/DDAC, with the presence of diffraction dots disposed on concentric rings, demonstrating the crystalline nature of AgCl NPs. Rings are ascribable, from inner to outer, to the [111], [200], [220], [311], [222] reflections [37,74].

In the case of AgCl/DDoAC (Fig. 6c and d), aggregated, spindle-shaped particles were observed. It is likely that the presence of DDoAC vesicles results in the formation of multi-shaped NPs. Furthermore, the AgCl/DDoAC 20 mM milky fluid was filtered using a 450 nm filter and subjected to TEM. Presumably, some of the AgCl NPs were retained in the filter, whereas some were retained in the milky fluid solution. Interestingly, rod and spindle shapes are observed, as shown in Fig. S4 (a, b, c).

### 3.3 Antimicrobial efficiency of AgCl NCs

**Fig. 7:**
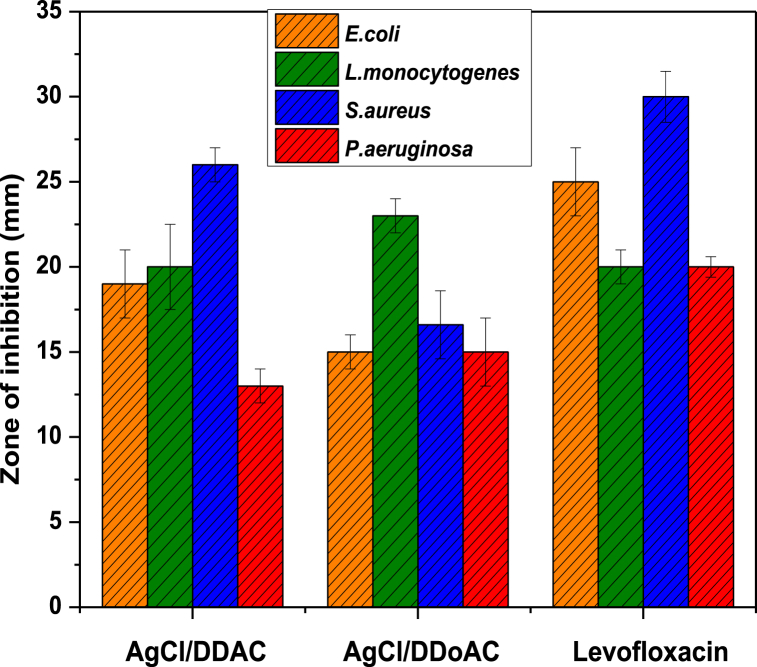
Antimicrobial susceptibility results from the agar well disk diffusion method. Graphical representative histogram of comparative inhibition zones between the conventional antibiotic levofloxacin and the noncolloidal solutions AgCl/DDAC and AgCl/DDoAC.

The disk diffusion method was used to evaluate the antimicrobial efficiencies of AgCl/DDAC and AgCl/DDoAC. In accordance with CLSI, with the aim of susceptibility and antimicrobial capacity, the diameter inhibition zone (DIZ) above 11 mm must be observed, depending on the antimicrobial concentration. The presence of a clear zone for all bacterial strains indicates that all AgCl/DDAC and AgCl/DDoAC non-colloidal solutions dropped in paper discs have antimicrobial properties. The DIZ around the antibiotic disc was not as clear as that of the synthesized nanocolloids. The results are presented in Fig. 7, with the inclusion of levofloxacin at a concentration of 5 μg showing an inhibition zone diameter of 25 ± 2 mm while the disk loaded with AgCl/DDAC at a concentration of 18 μg/mL (50 mM) reached 19 ± 2 mm for *E. coli.* The AgCl/DDAC NC solution with the same concentration tested showed the same antimicrobial efficiency as the antibiotic (19.0 ± 1.0 mm) with a diameter of 20 ± 3 mm for *L. monocytogenes* *46*, and a much higher inhibition with a 26.0 ± 1.0 mm zone of inhibition for *S. aureus*, indicating the same range as the conventional antibiotic levofloxacin with a diameter of 29.0 ± 1.0. The lower activity is noted for *P. aeruginosa* with an inhibition diameter of 13.0 ± 1.0 mm while the zone inhibition for the antibiotic is 20.0 ± 0.6 mm (Table 1).

**Table 1:** Determination of the diameter inhibition zone (DIZ) via agar diffusion test (ADIFF) against *E. coli ATCC* 25922, *Listeria monocytogenes* 46*, S. aureus ATCC* 29213, and *P. aeruginosa* 27853. The values are presented as the mean of three replicates ± standard deviation (SD). Agar diffusion test, CLSI M44 protocol.

Bacterial Strains	AgCl/DDAC ADIFF ± SD (mm)	DDAC ADIFF ± SD (mm)	AgCl/DDoAC ADIFF ± SD (mm)	DDoAC ADIFF ± SD (mm)	Levofloxacin 5 μg ADIFF ± SD (mm)
*E. coli* ATCC 25922	19 ± 2	15.3 ± 1.5	15 ± 1	11.0 ± 1.5	25 ± 2
*L. monocytogenes* 46	20 ± 2	12 ± 2	23 ± 1	15.0 ± 1.0	20 ± 1
*S. aureus* ATCC 29213	26 ± 1	17 ± 1	17 ± 2	11.0 ± 1.5	29.0 ± 1.5
*P. aeruginosa* ATCC 27853	13 ± 1	11.0 ± 0.5	15 ± 2	10 ± 1	19.0 ± 0.6

In contrast, the AgCl/DDoAC system exhibited comparable antimicrobial effectiveness to that of AgCl/DDAC NC. The antibiotic levofloxacin at a concentration of 5 μg, with a diameter of 19 ± 1 mm, was much lower than the inhibition zone of *L. monocytogenes* *46*, whereas the inhibition diameter of the AgCl/DDoAC-loaded disk was 23 ± 1 mm. Comparable results for other pathogenic bacteria are shown in the histogram above and in the inhibition zone values of AgCl NCs (Table 1). The DIZ values for the AgCl/DDoAC system showed that levofloxacin at a concentration of 5 μg had an inhibition zone ∼4.4% larger than nanoantimicrobials theirself. Foodborne pathogens of Gram-positive bacteria (*S. aureus* and *L. monocytogenes* *46*) and Gram-negative bacteria (*E. coli* and *P. aeruginosa*) were effectively treated at even lower concentrations by AgCl/DDAC and AgCl/DDoAC NC solutions.

### 3.4 Minimum inhibitory concentration (MIC)

In this study, modified minimum inhibitory concentration (MIC) determination was performed according to the Clinical Laboratory Standards Institute (CLSI, 2015, 2008) guidelines [75]. The lowest concentration of an antibacterial agent expressed in μg/mL, which defines the susceptibility or resistance of microbial strains, is known as the minimal inhibitory concentration (MIC) [76]. Similar to the bacterial growth inhibition zone in the qualitative method, the determination of MIC value serves as the basis for estimating the category and degree of susceptibility to the greatest importance in the optimization of targeted antibiotic therapy [77].

To test the antimicrobial capacity of the nanocolloids, four food pathogenic strains were analyzed. The MIC of AgCl/DDAC against *E. coli* ATCC 25922 strain was found to be 8.0 ± 0.7 μg/mL (Fig. S5 and Table 2). Susceptibility against other Gram-negative bacteria, *such as L. monocytogenes*, had a MIC value of 16 ± 2 μg/mL. The lowest possible minimum inhibitory concentration was observed against Gram-positive bacteria *S. aureus* ATCC 29213 at a concentration of only 2 μg/mL and for *P. aeruginosa* ATCC 27853 at 4.0 ± 0.7 μg/mL. The standard control of the surfactant alone was performed at a concentration of 256 μg/mL, which is the minimum inhibitory concentration of DDAC.

**Table 2:** Determination of MIC against *E. coli ATCC* 25922, *Listeria monocytogenes* 46*, S. aureus ATCC* 29213, and *P. aeruginosa ATCC* 27853. The values are presented as the mean of three replicates ± standard deviation (SD). Microdilution test, CLSI M7A9 protocol.

Bacterial Strains	AgCl/DDAC MIC ± SD (μg/mL)	DDAC MIC ± SD (μg/mL)	AgCl/DDoAC MIC ± SD (μg/mL)	DDOAC MIC ± SD (μg/mL)	Gentamicin MIC ± SD (μg/mL)
*E. coli* ATCC 25922	8.0 ± 0.7	>256	64 ± 1	>256	1.0 ± 0.5
*L. monocytogenes* 46	16 ± 2	>256	16.0 ± 0.5	>256	2.0 ± 0.6
*S. aureus* ATCC 29213	2.0 ± 0.5	>256	64.0 ± 0.6	>256	0.5 ± 0.6
*P. aeruginosa* ATCC 27853	4.0 ± 0.7	>256	128 ± 1	>256	2.0 ± 0.6

The efficiency of AgCl/DDoAC against bacterial Gram-positive strains resulted in higher MIC values, ranging from 64.0 ± 1.0 μg/mL against *S. aureus* and 128.0 ± 1.0 μg/mL for *P. aeruginosa*, compared to the Gram-negative strains, which showed much lower MIC values. The bacterial strain *E. coli* showed the same MIC value as *S. aureus*, whereas a significantly lower MIC value of 16.0 ± 0.5 μg/mL was apparent for *L. monocytogenes* as one of the most common food pathogenic antibiotic-resistant bacteria. The control DDoAC surfactant showed the same minimum inhibitory concentration as DDAC in all bacterial strains tested. The results are presented in Table 2, along with the correlation between MIC values. These values were confirmed by absorbance measurements and the viable cell count method. The antimicrobial efficacy of AgCl/DDAC and AgCl/DDoAc is compared with previously published AgCl based materials (Table S1).

### 3.5 Antibiofilm efficacy of AgCl nanocolloids

AgCl NCs solutions were examined for their antibiofilm activity against the same Gram-positive and Gram-negative bacteria by colorimetric XTT staining assay providing that in a concentration-dependent manner, inhibition of the biofilm formation was achieved by nanocolloids at all tested sub-MICs concentrations. The XTT assay detects the vitality of microorganisms in biofilms, permitting assessment of the efficacy of antimicrobial compounds [78,79]. This XTT reduction method makes direct comparisons between bacterial isolates because different strains metabolize XTT with different capabilities [80]. This is of unique importance, as other methods lack this specific correlation. Different abilities of the tested food pathogenic strains metabolized tetrazolium salts, with differences in XTT reduction rates. Living bacterial cells tending to form biofilm structures have reduced tetrazolium salt (2.3-Bis(2-methoxy-4-nitro-5-sulfophenyl)-2H-tetrazolium-5carboxanilide, XTT) in formazan as a hydro-soluble composite because of the NADH-mitochondrial dehydrogenases of the electron transport system (ETS) using redox pigment as an artificial electron acceptor that competes with oxygen.

**Fig. 8:**
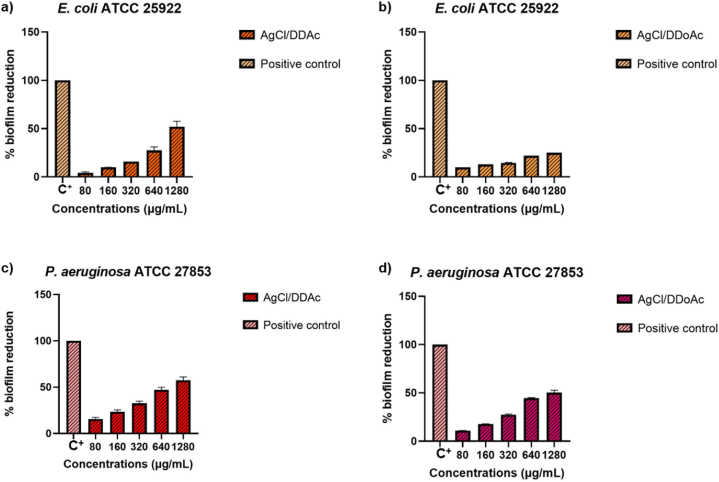
Percentage of biofilm reduction for Gram-negative bacterial strains exposed to different concentrations of silver chloride nanocolloids evaluated through the XTT assay; Antibiofilm behaviour of (a) AgCl/DDAC nanocolloids against *E. coli* ATCC 25922, (b) AgCl/DDoAC nanocolloid against *E. coli* ATCC 25922 biofilms, (c) AgCl/DDAC nanocolloids against *P. aeruginosa* ATCC 27853, and (d) AgCl/DDoAC nanocolloid against *P. aeruginosa* ATCC 27853.

The evaluation of microbial biofilm reduction after exposure to different concentrations of AgCl/DDAC and AgCl/DDoAC is expressed as a percentage, as shown in Fig. 8. The decrease is attributed to the inhibitory activity of the two compounds against biofilm formation. The percentage represents the mass reduction, as evidenced by formazan reduction indicated by the absence of the color.

Viable biofilm growth was estimated using the XTT reduction assay and the impact of biofilm therapy with the addition of AgCl NCs. Biofilms were formed 24 h after attachment to the bottom of the microtiter plate before the addition of the antimicrobial agents AgCl/DDAC and AgCl/DDoAC. The detected percentage of biofilm reduction was observed with differences in the steepest part of the dose-response relationship. Considering the colorimetric measurements, the endpoint determination varied with the concentration needed for a higher percentage of biofilm inhibition. The reference strain of positive control bacteria was compared to that of treated biofilm samples by measuring the absorbance at 490 nm. Following treatment with 1280 μg/mL AgCl/DDAC, the growth of *E. coli* ATCC 25922 biofilm was reduced by (50.0 ± 0.5)%, while the concentrations of 320 and 640 μg/mL of the active compound showed even higher biofilm reduction ability by (60.0 ± 1.0)% and (70.0 ± 0.5)%, respectively. In the case of the smallest MIC concentrations tested (160 μg/mL and 80 μg/mL), the reduction of *E. coli* biofilm was (80.0 ± 0.5)% and (90.0 ± 0.3)%, respectively (Fig. 8a). Regarding the organic system of AgCl/DDoAC/IPA, the growth of *E. coli* ATCC 25922 was reduced similarly to AgCl/DDAC at all MIC concentrations, which is expected as they belong to the same nanocolloidal type of quaternary ammonium chlorides. Biofilm reduction of (65.0 ± 0.5)% was achieved for 640 ± 1 μg/mL AgCl/DDoAC, which was 5 % higher than that achieved with the DDAC system (Fig. 8b).

Regarding the other Gram-negative bacteria, *P. aeruginosa* ATCC 27853, biofilm inhibition efficiency was achieved again for the minimum concentration tested at (80.0 ± 0.5)% for AgCl/DDAC and (85.0 ± 0.2)% for treated solutions of AgCl/DDoAC (Fig. 8c and d). A periodical decrease in biofilm reduction is noted in the scale of lower concentrations for a grade of 10 % difference. The reduction percentages at 1280, 640, 320, and 160 μg/mL were (50.0 ± 0.5)%, (60.0 ± 0.7)%, for 320 μg/mL is (70.0 ± 0.5)% and for 160 μg/mL is (80.0 ± 1.0)%, respectively. The robust nanocolloidal system containing silver chloride nanoparticles potentially inhibited the biofilm formation of *P. aeruginosa ATCC 27853*.

**Fig. 9:**
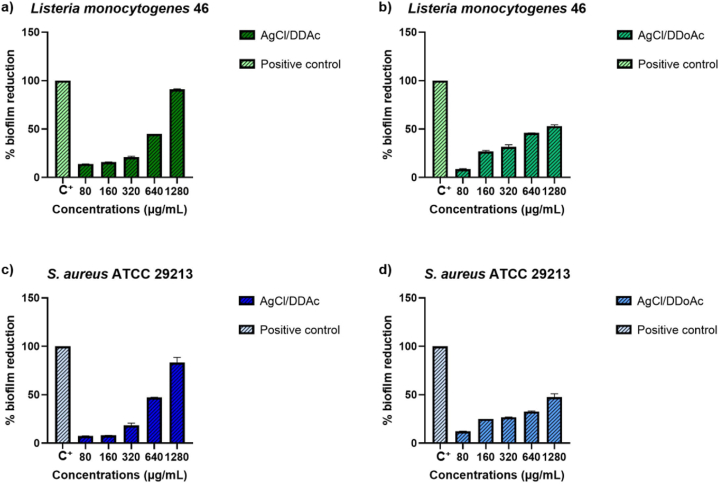
Percentage of biofilm reduction for Gram-positive bacterial strains exposed to different concentrations of silver chloride nanocolloids evaluated by the XTT assay: Antibiofilm behaviour of (a) AgCl/DDAC nanocolloids against *L. monocytogenes* 46, (b) AgCl/DDoAC nanocolloid bioactivity against *L. monocytogenes* 46 biofilms, (c) AgCl/DDAC nanocolloids against *Staphylococcus aureus* ATCC 29213, and (d) AgCl/DDoAC nanocolloids against *Staphylococcus aureus* ATCC 29213 biofilms.

In anticipation of Gram-positive bacterial strains, the efficiency of *L. monocytogenes* 46 biofilm inhibition increases at low antimicrobial concentrations. The action of high concentrations of AgCl nanoparticles provokes intense coagulation of cell wall proteins, together with the external capsular matrix, which becomes an obstacle to the subsequent destruction of the biofilm [81,82]. Therefore, lower concentrations of *L. monocytogenes* 46 were more effective. The maximum tested concentration of 1280 μg/mL displayed the smallest biofilm reduction percentage (10.0 ± 0.5)%, demonstrating that the optimal concentration of AgCl/DDAC for MIC values inhibited the majority of *L. monocytogenes* 46 biofilm formation. These concentrations showed the best inhibition behavior of reduction, similar to *E. coli* biofilms, with a high increase in biofilm inhibition at (65.0 ± 0.7)% for 640 μg/mL, (70.0 ± 0.5)% for 320 μg/mL, and (80.0 ± 0.5)% for 80 μg/mL and 160 μg/mL noncolloidal solutions (Fig. 9a).

Concerning the influence of green, scalable nanocolloids on Gram-positive bacterial biofilms, the XTT susceptibility test for antibiofilm activity of AgCl/DDoAC against *L. monocytogenes* 46 biofilms formation showed inhibition activity at the maximum concentration from 50% biofilm reduction, which was 15% less than the same concentration for the AgCl/DDAC system (Fig. 9b). The smallest tested concentrations of AgCl/DDoAC from 80 μg/mL showed (85.0 ± 1.0)% and 5% more biofilm reduction ability than silver chloride/DDAC nanocolloids, except for the other concentrations of 160 and 320 μg/mL, which showed lower inhibition activity compared to the AgCl/DDAC noncolloidal solution. The AgCl/DDAC system also demonstrated optimal antibiofilm performance for the isolated *S. aureus* ATCC 29213 strain (Fig. 9c). The minimum tested concentration (80 μg/mL) reduced biofilm formation by (90.0 ± 0.5)%, whereas the same concentration of AgCl/DDoAC resulted in only (80.0 ± 0.7)% biofilm reduction. Similar behavior was observed for 160 μg/mL AgCl/DDAC, which decreased biofilm formation by (90.0 ± 1.2)%, although the same concentration of AgCl/DDoAC nanocolloids gave 70% biofilm inhibition efficiency, while the other concentrations (640 and 1280 μg/mL) showed 20% less biofilm reduction for AgCl/DDAC compared to the AgCl/DDoAC system (Fig. 9d). In the case of AgCl/DDAC it is observed that at a concentration of 320 μg/mL, (85.0 ± 0.5)%, biofilm reduction was achieved. Conversely, for AgCl/DDoAC at same concentration, biofilm inhibition was lower (65.0 ± 0.3)%.

The XTT assay and microtitre plate models with food pathogenic bacteria i are valuable tools for investigating the effectiveness of bioactive compounds such as AgCl/DDAC and AgCl/DDoAC against biofilms. The experimental methodology facilitated the statistical assessment of triplicate measurements' performance.

### 3.6 Determination of vitality of cells by CFU counting

Viable cell counting is a well-known standard quantification method for separating individual cells on an agar plate by growing colonies of bacterial cells, consequently differentiating dead cells from living biomass and the quantification of vital cells [83,84]. Enumeration in pure liquid cultures was performed, as the optical density (OD) must be measured prior to plating of microbial strains for CFU counting. To test the viability reduction of the foodborne pathogenic bacteria tested, the means of the optical density values (OD = 600 nm) were evaluated for the noncolloidal solutions deposited onto glass and Si wafer coverslips (Fig. S6).

The Gram-negative bacterium *E. coli* ATCC 25922 showed a reduction in viability after treatment with silver chloride nanocolloids, as evidenced by a decrease in the percentage of OD reduction to 45% for AgCl/DDAC and 30 % AgCl/DDoAC, whereas the surfactant itself showed less efficient reduction (Fig. S6a). Surprisingly, DDoAC 50 mM itself showed more viability reduction than DDAC 50 mM for 10 % more. Another problematic Gram-negative bacterium, *P. aeruginosa* 27853, showed less inhibitory activity than S*. aureus* 29213 (Fig. S6d). The coverslips treated with AgCl/DDAC and tested against *P. aeruginosa* 27853 have also proven the inhibition by an optical density reduction of ∼45 %, whereas for AgCl/DDoAC, the maximum reduction was 30%. In parallel with previous results, surfactants alone showed a much lower reduction, more likely ∼10–15%. Gram-positive bacteria differ from Gram-negative bacteria in the chemical and physical properties of their cell walls [85]. Considering this, it has been observed the highest efficiency of AgCl/DDAC-deposited coverslips on the viability decrease of Gram-positive bacteria. The viability of the S*. aureus* 29213 strain was reduced by 100%, indicating a complete and successful inhibition of bacterial growth (Fig. S6c). The other non-colloidal coverslips treated with AgCl/DDoAC displayed a 60% reduction in viability against the same bacterial strain. The control of surfactants showed 65% inhibition of DDAC itself and only a 10% reduction in viability by DDoAC. Interestingly, the last Gram-positive bacterium *Listeria monocytogenes* 46, was shown to reduce cell viability by coverslips treatment with AgCl/DDAC and the percentage of biofilm reduction was 95%. This represents the most significant viability reduction achieved so far, demonstrating a notable bacteriostatic effect. On the other hand, the AgCl/DDoAC system exhibited only a 10% reduction in the optical density value. Similar to the behavior observed with DDAC as a control, DDoAC showed a slight decrease with no significant reduction (Fig. S6b).

In general, it can be concluded that the synthesized AgCl NCs strongly reduced the viability of bacterial cells for both Gram-negative and Gram-positive bacteria.

Table 3 shows the CFU counts of the four bacterial strains after treatment with AgCl/DDAC and AgCl/DDoAC. The CFU/mL was used to quantify the viable cells and showed very low values of inoculum detected after treatment with AgCl/DDAC compound for each bacterial strain and with the smallest number of colonies for *P. aeruginosa* ATCC 27853. The other non-colloidal system of AgCl/DDoAC also distinguished a low number of CFU counts manifesting the efficient quantification of viable cells as a result of antimicrobial activity.

**Table 3:** Colony-forming unit (CFU) counts of *E. coli*, *Listeria monocytogenes46*, *S. aureus*, and *P. aeruginosa* for the serially diluted 10 folds of cultured suspension with nanocolloid-treated coverslips. The values are presented as the mean of three replicates ± standard deviation (SD).

Colony forming unit (CFU/mL) ± SD					
Bacterial Strains	Positive control	DDAC	AgCl/DDAC	DDoAC	AgCl/DDoAC
*E. coli* ATCC 25922	1600 ± 2	1200 ± 1	80 ± 1	1400 ± 2	300 ± 1
*L. monocytogenes* 46	1750 ± 1	1600 ± 1	100 ± 2	1600 ± 1	1000 ± 2
*S.aureus* ATCC 29213	1500 ± 2	150.0 ± 0.5	60 ± 2	1300.0 ± 0.5	290 ± 1
*P. aeruginosa* ATCC 27853	1200 ± 4	1000 ± 2	40 ± 2	950 ± 1	260 ± 1

## 4 Conclusions

Rapid, large-scale production of antimicrobial agents, safe technology, cost-effectiveness, easily scalable, and versatility is often unnoticed. In this study, we have reported a standard argentometric titration to produce versatile, scalable, fast, safe, and robust AgCl/quaternary ammonium compound (QACs) colloidal NAMs. The possibility of a large-scale production of the colloidal suspensions was explored through the application of a peristaltic pump.

The use of various types of biosafe QACs and a wide range of solvents, including aqueous and organic ones, makes this system green and versatile. Moreover, the presence of intrinsically insoluble AgCl could increase the level of control over the extent and rate of generation of bioactive species, by providing a thermodynamically-controlled release of Ag^+^ ions. Detailed analytical characterization, including UV–Vis, TEM, SAED, FTIR, and XPS, confirmed the formation of core-shell AgCl NPs in the colloidal state. Moreover, the DLS size distribution and hydrodynamic diameter of AgCl NPs were observed for both AgCl/DDAC and AgCl/DDoAC nanocolloidal systems. A sharp and narrow size distribution and highly positive zeta potential value were obtained for the AgCl/DDAC colloids. Strong antimicrobial and antibiofilm effects of AgCl NCs have been observed. Nanocolloids inhibited microbial growth and biofilm formation of different foodborne pathogenic bacterial species, namely *Staphylococcus aureus* ATCC 29213*,* Listeria monocytogenes 46, *Escherichia coli* ATCC 25922, and *Pseudomonas aeruginosa* ATCC 27853. Foodborne Gram-positive and Gram-negative bacteria were effectively inhibited by low MICs of AgCl/DDAC and AgCl/DDoAC NC solutions. Both candidates were found to be potential inhibitors of food-pathogenic bacterial biofilm formation, as investigated by the colorimetric XTT staining assay, through the evaluation of microbial biofilm reduction after exposure to various sample concentrations. The low CFU counts indicated that our nanocolloidal material strongly reduced the viability of bacterial cells for both Gram-negative and Gram-positive bacteria. This raises the possibility of treating antibiotic-resistant infections by biofilm-forming microbes. The proposed systems are mild and active against a wide range of pathogenic bacteria and biofilms. The results of this study present prospects for developing industrial products based on stable AgCl nanocolloids that can be incorporated as active additives into food packaging systems to increase the shelf life of dairy food products and ensure prolonged freshness on an industrial scale.

## Intellectual property

We confirm that we have given due consideration to the protection of intellectual property associated with this work and that there are no impediments to publication, including the timing of publication, with respect to intellectual property. In so doing we confirm that we have followed the regulations of our institutions concerning intellectual property.

## Research ethics

We further confirm that any aspect of the work covered in this manuscript that has involved human patients has been conducted with the ethical approval of all relevant bodies and that such approvals are acknowledged within the manuscript.

IRB approval was obtained (required for studies and series of 3 or more cases).

Written consent to publish potentially identifying information, such as details or the case and photographs, was obtained from the patient(s) or their legal guardian(s).

## Authorship

The International Committee of Medical Journal Editors (ICMJE) recommends that authorship be based on the following four criteria

## Contact with the editorial office

The Corresponding Author declared on the title page of the manuscript is:

## CRediT authorship contribution statement

**Diellza Bajrami:** Writing – original draft, Methodology, Investigation, Formal analysis, Data curation, Conceptualization. **Syed Imdadul Hossain:** Writing – original draft, Methodology, Investigation, Formal analysis, Data curation, Conceptualization. **Alexia Barbarossa:** Methodology, Formal analysis, Data curation. **Maria Chiara Sportelli:** Writing – review & editing, Writing – original draft, Supervision, Methodology, Investigation, Formal analysis, Data curation. **Rosaria Anna Picca:** Writing – review & editing, Supervision, Methodology, Data curation. **Luigi Gentile:** Methodology, Formal analysis, Data curation. **Francesco Mastrolonardo:** Formal analysis, Data curation. **Antonio Rosato:** Writing – review & editing, Supervision. **Alessia Carocci:** Writing – review & editing, Supervision. **Nicola Antonio Colabufo:** Writing – review & editing, Supervision. **Boris Mizaikoff:** Writing – review & editing, Supervision, Resources, Project administration, Funding acquisition. **Nicola Cioffi:** Writing – review & editing, Supervision, Resources, Funding acquisition.

## Declaration of competing interest

The authors declare that they have no known competing financial interests or personal relationships that could have appeared to influence the work reported in this paper.
