# Supplementary material for: A scalable route to quaternary ammonium-functionalized AgCl colloidal antimicrobials inhibiting food pathogenic bacteria and biofilms
Source: Heliyon. 2024 Feb 1;10(3):e25260. doi: 10.1016/j.heliyon.2024.e25260 (PMC10847915; doi:10.1016/j.heliyon.2024.e25260)
Supplement: Multimedia component 1 [file mmc1.docx]

Supporting Information (SI)

**A scalable route to quaternary ammonium-functionalized AgCl colloidal antimicrobials inhibiting food pathogenic bacteria and biofilms**

Diellza Bajrami^a,1^, Syed Imdadul Hossain^b, c,1^, Alexia Barbarossa^d^, Maria Chiara Sportelli^b^, Rosaria Anna Picca^b,c^, Luigi Gentile^b,c^, Francesco Mastrolonardo^e^, Antonio Rosato^d^, Alessia Carocci^d^, Nicola Antonio Colabufo^d,e^, Boris Mizaikoff ^a,f,^*** and Nicola Cioffi ^b,c,^***

^a^ Institute of Analytical and Bioanalytical Chemistry, Ulm University, Ulm, Germany

^b^ Chemistry Department, University of Bari “Aldo Moro”, Bari, Italy

^c^ CSGI (Center for Colloid and Surface Science) c/o Dept. Chemistry, Bari, Italy

^d^ Department of Pharmacy-Drug Sciences, University of Bari “Aldo Moro”, Bari, Italy

^e^ Biofordrug srl, University of Bari “Aldo Moro,” Triggiano, Bari, Italy.

^f^ Hahn-Schickard, Institute for Microanalysis Systems, Ulm, Germany

*Corresponding authors. B. M: [boris.mizaikoff@uni-ulm.de](file:///C:\Users\rosan\Documents\Picca_Dell\Hossain\AgCl_DDAC_DDoAC\boris.mizaikoff@uni-ulm.de); N. C: [nicola.cioffi@uniba.it](file:///C:\Users\rosan\Documents\Picca_Dell\Hossain\AgCl_DDAC_DDoAC\nicola.cioffi@uniba.it)

^1^ Both authors equally contributed to this work**.**

Number of pages: 10

**Antibiofilm activity test**

**Formazan formation reaction from XTT**

Formazan production is proportional to the initial XTT concentration (according to the saturation law) and cell biomass vitality. The reduction in formazan formation indicates cell death due to the products used. Menadione was added to the mixture because it acts as a catalyzing agent for the oxidation-reduction reaction. After the contact period with the drug, the media was removed from each well, and the supports were washed with 1 mL of PBS. The biofilms were then plunged in 2 mL of PBS with the addition of 180μl of XTT solution (1 mg/mL) and menadione (0.4 mM) at a ratio of 6:1. This solution was prepared by solubilizing XTT in sterile water (obtained by filtration) and menadione in dimethyl sulfoxide. The plate with supports was incubated for 2 h at 37°C. The initial color of the XTT-menadione mixture was yellow. The formation of formazan leads to a change in the color of the solution from yellow to orange. The intensity of formazan formation was highlighted by a more intense orange color. This indicates a higher vitality of bacterial cells owing to their mitochondrial activity. At the end of the 2 hours, the plate is read at microplate spectrophotometer (Perkin-Elmer Wallac Victor3 Microplate reader) at a 490 nm wavelength, where there is the maximum Formazan absorbance peak. It is a method little used in international antibiofilm testing, although it is the most useful, thanks to the fact that it needs volumes well below 24-wells method, going from milliliters to microliters and producing a considerable cost reduction.

**Culture phase:** Cryovials containing bacterial strains were defrosted inside an oven at 37°C, and each cryovial was poured into a specimen containing TSB media + glucose 2%. The mixture was then stirred at 37°C for 24 h. After 24 h, the cell suspension was calibrated at λ of 625 nm with DO between 0.08 and 0.10 nm. Then, 2-3 colonies of the suspension are taken and 4.5 mL of MHB are suspended again; after having "vortexed" for some seconds, calibration is performed. Once the correct degree of absorbance was obtained, 100 μL was collected and placed in a second tube containing 9.9 mL of TSB, thus obtaining a dilution of 1:100. The bacterial suspension was "vortexed, " and from this last dilution, 200 µL of multichannel was collected and placed in single required wells of a flat-bottomed plate; the last line represents the control wells. Plates were incubated aerobically on a horizontal shaker for 4 h at 37 °C. After the suspension was gently removed and replaced with sterile medium, the plate was incubated for 24 h at 37 °C to obtain mature biofilms.

**Maturation phase:** After the time necessary for bacterial cells to attach to the plate bottom, the inoculum contained in each well was removed, and the washing phase with 100 μL of PBS was performed, paying attention to not touching the fully-grown biofilm and collecting the possible residuals. Then, 200 μL of the substance to be tested, previously suitably diluted, was added to every well, except the control.

*Colorimetric method XTT:* After the contact period with the drug, the medium was removed from the well, washed with PBS, and placed upside down in an oven at 37°C for 20 min. In this manner, 40 μL of the XTT solution was added. The plate was then incubated in the dark for two hours at 37°C with stirring. After two hours, 150 μL of the supernatant was collected and placed on a new plate, paying attention to the correct position of the wells. The plate was suitably covered, and the OD was verified at 490 nm. The evaluation of absorbance revealed the action of the product against the biofilm compared to the positive control of growth.

**Direct band gap calculation**

The optical band gap (ɛ_g_) was calculated using the Tauc equation:^S1^

εhv = c(hv-ɛ_g_)^n^

where ɛ is the molar absorption coefficient (given by 2.302 Acm^-1^,^S2^ where A is absorbance and quartz cells of 1 cm path length), ɛ_g_ is the optical band gap, hv is the photon energy, c is band tailing parameter, and n = 1/2 for direct band gap.^S3^

**Fig. S1.** Tauc plots for the determination of band gap of AgCl/DDAC and AgCl/DDoAC.


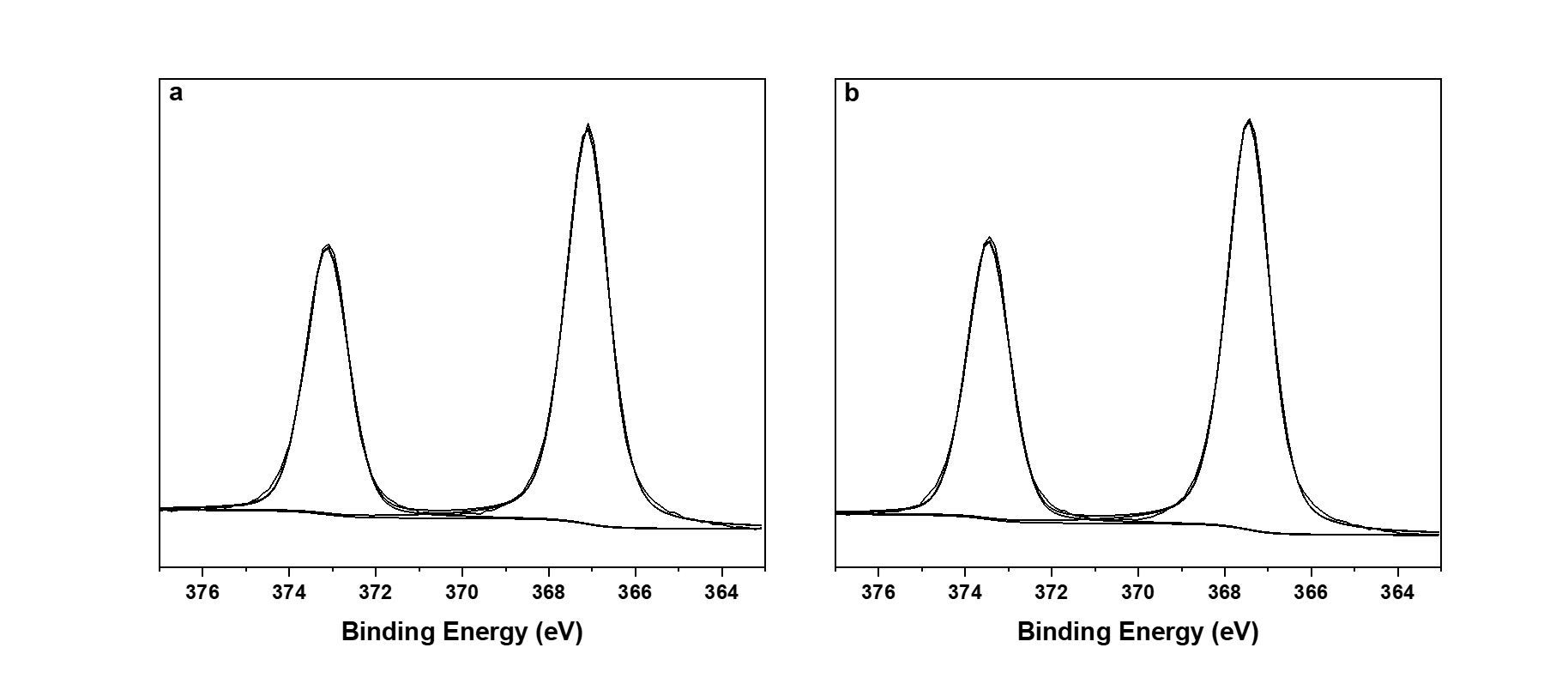


**Fig. S2** Ag3d spectra of (a) AgCl/DDAC and (b) AgCl/DDoAC.


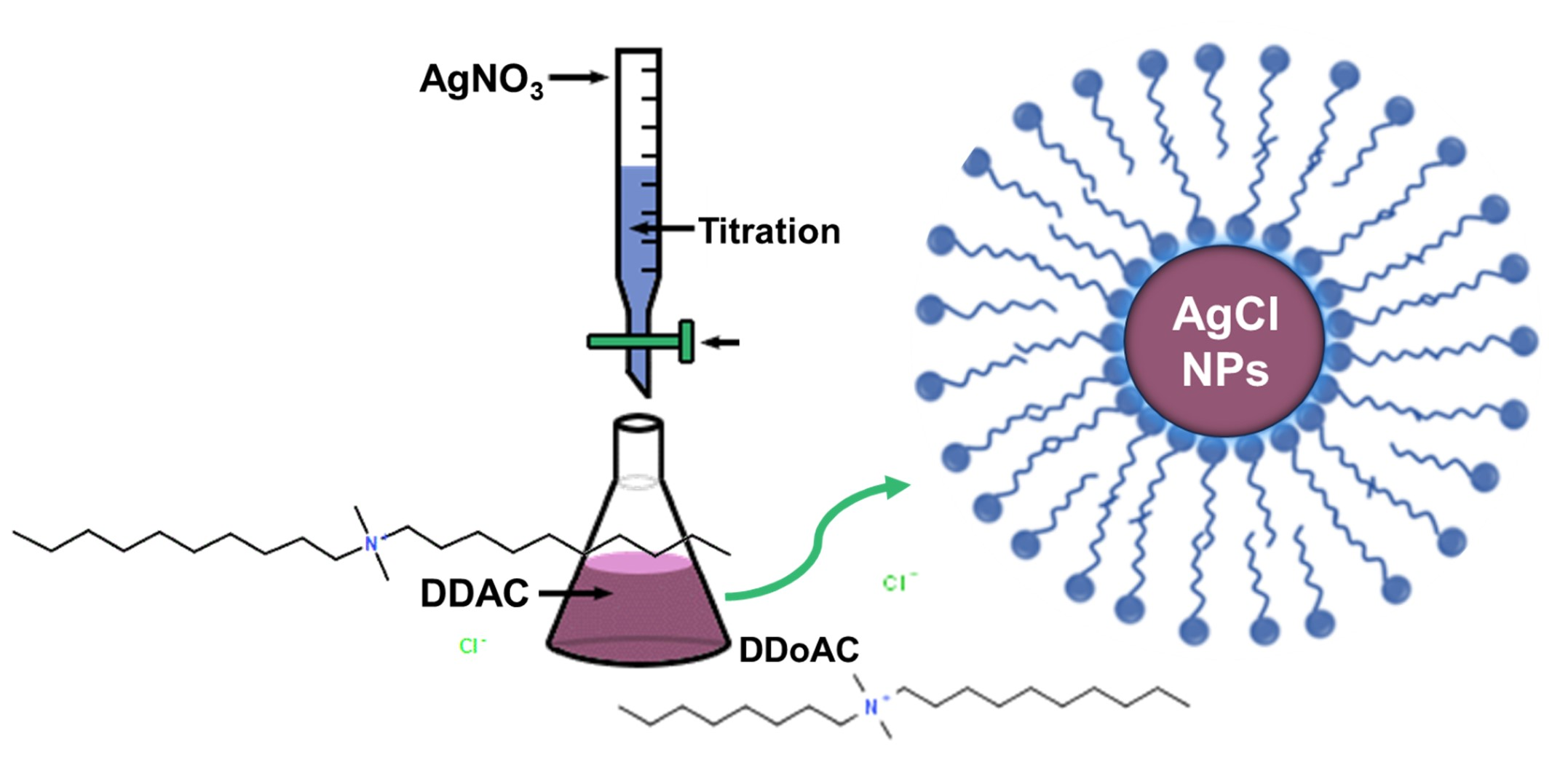


**Fig. S3** Schematic representation for AgCl NPs stabilized by DDAC^+^ and DDoAC^+^ molecules.


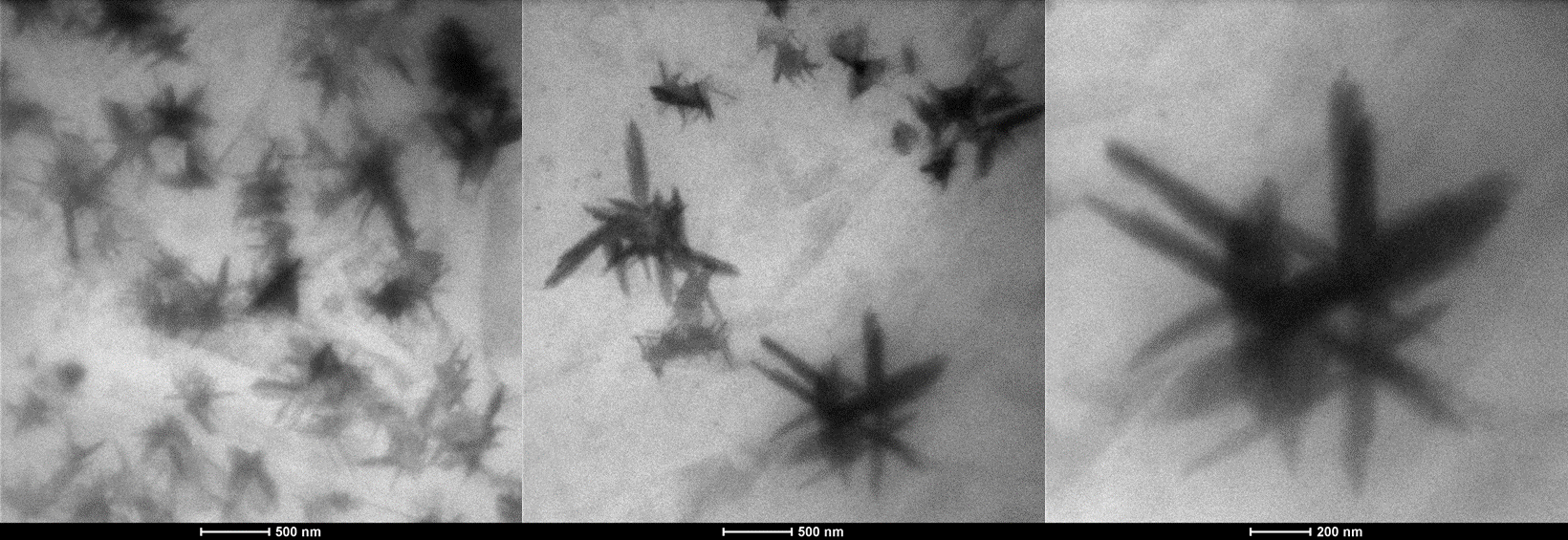


(a)

(b)

(c)

**Fig. S4** (a), (b), (c) Typical TEM images of AgCl/DDoAC (450nm filtered).

**
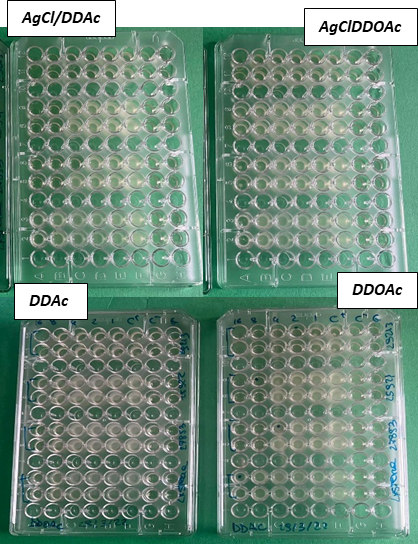
**

**Fig. S5.** Determination of minimum inhibitory concentration


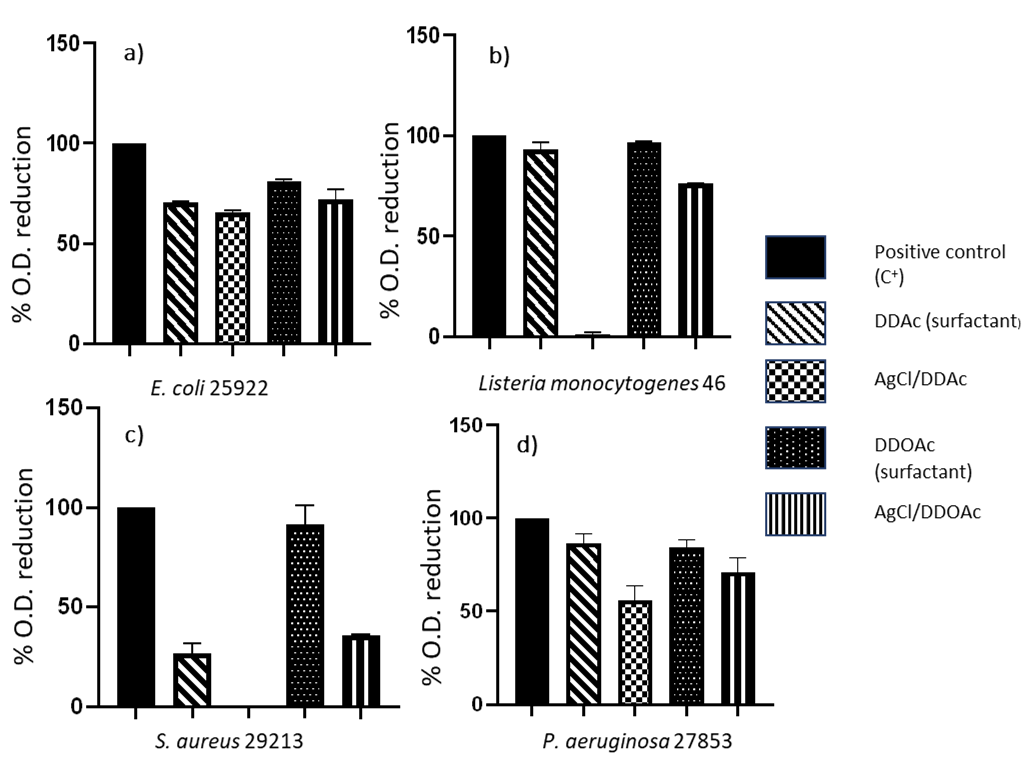


**Fig. S6**. Percentage of viability reduction of microbial strains tested was assessed by optical density (OD=600 nm) evaluation. The bacterial suspension strains, (a) *E. coli* ATCC 25922, (b) *Listeria monocytogenes* 46, (c) S*. aureus* 29213, and (d) *P. aeruginosa* 27853, were exposed for 24 h to coverslips with dried DDAC, AgCl/DDAC, DDoAC, and AgCl/DDoAC on top of the surface.

**Table S1.** The antimicrobial efficacy of AgCl nanoparticles synthesized in this study comparable to similar materials from literature.

| Antimicrobial agent | Bacterial inhibition | Strains tested | References |
| --- | --- | --- | --- |
| AgCl/DDAC | Diameter of inhibitory zones 13–26 mm for 2-16 μg/mL (MICs) | *E. coli* ATCC 25922  *L. monocytogenes* 46  *S. aureus* ATCC 29213  *P. aeruginosa* ATCC 27853 | Present study |
| AgCl/DDoAC | Diameter of inhibitory zones 15–23 mm for 16-128 μg/mL (MICs) | *E. coli* ATCC 25922  *L. monocytogenes* 46  *S. aureus* ATCC 29213  *P. aeruginosa* ATCC 27853 | Present Study |
| Biosynthesized Ag/AgCl NPs using *M. citrifolia* extract | Diameter of inhibitory zones from 12 up to 25 mm | [*Bacillus subtilis*](https://www.sciencedirect.com/topics/biochemistry-genetics-and-molecular-biology/bacillus-subtilis),  [*Bacillus cereus*](https://www.sciencedirect.com/topics/biochemistry-genetics-and-molecular-biology/bacillus-cereus),  [*Staphylococcus aureus*](https://www.sciencedirect.com/topics/medicine-and-dentistry/staphylococcus-aureus) and  [*Pseudomonas aeruginosa*](https://www.sciencedirect.com/topics/biochemistry-genetics-and-molecular-biology/pseudomonas-aeruginosa) | [S4] |
| Biosynthesized Ag/AgCl NPs using *Chara* algae extract | Diameter of inhibitory zones up to 18.7 mm, 13–68μg/mL (MICs) | *Staphylococcus aureus*,  *Escherichia coli*,  *Klebsiella pneumonia*, and  *Pseudomonas aeruginosa* | [S5] |
| AgCl NPs based on LDH, LDHa and LDHb | 2.8–8.5 μg/mL (MICs) | *S. aureus (ATCC 29213),*  *S. epidermidis (ATCC 12228),*  *P. aeruginosa (ATCC 15692) and one fungal species Candida albicans (CAF2/1)* | [S6] |
| Ag/AgCl NPs synthesized from *Fusarium oxysporum* | Diameter of inhibitory zones up to 6.0 mm, 10.52 μg/mL (MIC) | *Serratia mascescens, Klebsiella pneumoniae carbapenemase-KPC strains and*  *E. coli 25922* | [S7] |
| *Agrimonia pilosa* extract-mediated Ag-AgCl NPs | Diameter of inhibitory zones 14–17, 33 mm for 40-90 μg/mL (MICs) | *Bacillus cereus*  *Staphylococcus aureus*  *Staphylococcus saprophyticus*  *Listeria monocytogenes*  *Pseudomonas putida* | [S8] |

**References**

[S1] Mukherjee, I.; Dinda, G.; Ghosh, S.; Moulik, S. P. (2012). Synthesis, characterization, and applications of microheterogeneous-templated CdS nanodispersions. *Journal of Nanoparticle Research*. 14, 997

[S2] Hamed, A. S.; Abd El Aal, S.A.; Al- Abyad, M.; Seddik, U.; Mansour, N.A. (2020). Gamma-Ray Induced Optical and Structural Modifications in PADC and Makrofol Nuclear Detectors. *Journal of Radiation and Nuclear Application*. 5 (3), 193-199

[S3] Majumder, S.; Naskar, B.; Ghosh, S.; Lee, C-H.; Chang, C-H.; Moulik, S. P.; Panda, A. K. (2014). Synthesis and characterization of surfactant stabilized nanocolloidal dispersion of silver chloride in aqueous medium. *Colloids and Surfaces A: Physicochemical and Engineering Aspects.* 443, 156–163

[S4] Ashok Kumar, D., Palanichamy, V., Roopan, S.M., 2014. Photocatalytic action of AgCl nanoparticles and its antibacterial activity. Journal of Photochemistry and Photobiology B: Biology 138, 302–306. https://doi.org/10.1016/j.jphotobiol.2014.06.011

[S5] Hassan, K.T., Ibraheem, I.J., Hassan, O.M., Obaid, A.S., Ali, H.H., Salih, T.A., Kadhim, M.S., 2021. Facile green synthesis of Ag/AgCl nanoparticles derived from Chara algae extract and evaluating their antibacterial activity and synergistic effect with antibiotics. Journal of Environmental Chemical Engineering 9, 105359. https://doi.org/10.1016/j.jece.2021.105359

[S6] Nocchetti, M., Donnadio, A., Ambrogi, V., Andreani, P., Bastianini, M., Pietrella, D., Latterini, L., 2013. Ag/AgCl nanoparticle decorated layered double hydroxides: synthesis, characterization and antimicrobial properties. J. Mater. Chem. B 1, 2383–2393. https://doi.org/10.1039/C3TB00561E

[S7] Picoli, S., Durán, M., Andrade, P., Duran, N., 2016. Silver nanoparticles/silver chloride (Ag/AgCl) synthesized from Fusarium oxysporum acting against Klebsiella pneumouniae carbapenemase (KPC) and extended spectrum beta-lactamase (ESBL). Frontiers in Nanoscience and Nanotechnology 2, 107–110. https://doi.org/10.15761/FNN.1000117

[S8] Patil, M.P., Seo, Y.B., Kim, G.-D., 2018. Morphological changes of bacterial cells upon exposure of silver-silver chloride nanoparticles synthesized using Agrimonia pilosa. Microbial Pathogenesis 116, 84–90. https://doi.org/10.1016/j.micpath.2018.01.018
